# Sponge Galectins: Structure, Evolution, and Function

**DOI:** 10.1021/acsomega.5c13334

**Published:** 2026-04-01

**Authors:** Andressa Rocha de Oliveira Sousa, Jessica de Assis Duarte Ferreira da Ponte, Alexandre Holanda Sampaio, Celso Shiniti Nagano, Rômulo Farias Carneiro

**Affiliations:** Laboratório de Biotecnologia Marinha−BioMar−Lab, Departamento de Engenharia de Pesca, Universidade Federal do Ceará, Campus do Pici s/n, Bloco 871, 60440-970 Fortaleza, Ceará, Brazil

## Abstract

Marine organisms display remarkable chemical diversity and are a prolific source of bioactive molecules. Among these compounds are lectins, proteins or glycoproteins that have specific and reversible binding to carbohydrates. However, lectins do not alter the properties of their ligands and are not derived from adaptive immune responses. In animals, lectins are structured into families. Galectins constitute a well-defined family characterized by their affinity for β-galactosides and the presence of a conserved carbohydrate recognition domain (CRD). These proteins are widespread across the animal kingdom, from sponges to vertebrates, and are involved in diverse physiological processes, including cell recognition, signaling, and immune modulation. Sponges (phylum Porifera), as the earliest metazoans, have emerged as valuable models for understanding the structural evolution and functional diversification of lectins. Although several sponge lectins have been isolated, only a limited number have been fully characterized at the molecular level. Within this group, galectins are particularly intriguing due to their structural variability and distinctive carbohydrate-binding properties compared to their vertebrate counterparts. To date, eight sponge species have been reported to produce galectins, yet only three-dimensional (3D) structures have been experimentally determined. This scarcity of structural data limits our understanding of the evolutionary and functional aspects of these molecules. In this review, we summarize current advances in the structural and biochemical characterization of sponge galectins, compare their primary and predicted tertiary structures with those of vertebrate galectins, and discuss their emerging biotechnological potential.

## Graphical abstract

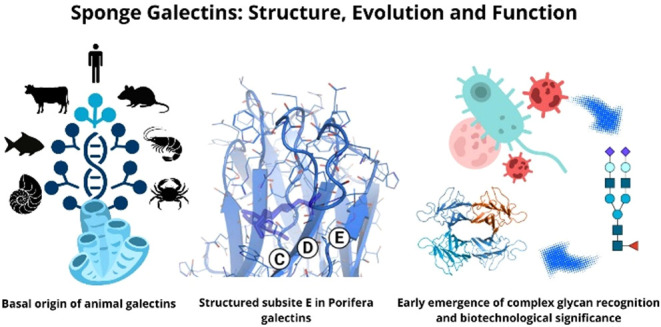

## 1 Introduction

Sponges, belonging to the Porifera phylum, represent the base lineage of the metazoan kingdom and are considered the oldest animal phylum on the planet.^1^ The great environmental variations, which sponges, and their associated microorganisms were able to support, leading to the accumulation of diverse bioactive compounds associated with their defense mechanisms, making these organisms prolific sources of natural marine products.^2^

The metabolites produced by sponges include alkaloids, terpenoids, proteins, polyketides, steroids, macrolides, nucleosides, fatty acids and peroxides.^3,4^ The structural diversity of these compounds underlies their wide range of biological activities, such as antitumor, antiviral, anti-inflammatory, immunosuppressive, antibiotic, antioxidant, hypocholesterolemic, neurosuppressive, muscle relaxant, antimalarial antifouling and antifungal.^3,5^

Due to the diversity of bioactive substances found in sponges, increasing interest has arisen in recent years in the chemical and pharmacological characterization of these organisms.^5^ Among the molecules that gained prominence are lectins, defined as not immunoglobulins proteins or glycoproteins that specifically and reversibly recognize carbohydrates without altering the structure of their ligands.^6,7^

Lectins are widely distributed across living organisms, from bacteria and fungi to plants and all animal phyla.^8^ In animals, these proteins perform diverse functions, many of them associated with innate immunity, where they act as pattern recognition receptors (PRRs) by recognizing pathogen-associated molecular patterns (PAMPs) through their carbohydrate recognition domains (CRDs).^9,10^ By binding specific glycans on microbial or abnormal cell surfaces, lectins promote agglutination, opsonization, complement activation, and pathogen clearance.^11^

These activities rely on protein–carbohydrate interactions, as cell-surface glycosylation patterns encode biological information that lectins decode as part of the so-called “glycocode”.^9,12^ Therefore, structural and glycobiological studies are essential to understand lectin function and their biotechnological potential. In this review, we focus on the structural features of sponge galectins, comparing them with galectins from other animal groups to provide insights into their evolution and biological roles.

## 2 Galectins

Galectins are β-galactoside–binding lectins widely distributed in fungi and throughout the animal kingdom. Initially termed “S-type lectins” because the activity of the first described members depended on reducing conditions, this designation was later abandoned, as redox sensitivity is not universal in the family.^13^

Galectins are synthesized in the cytosol as nonglycosylated proteins lacking signal peptides, yet they can be detected at the cell surface, in the extracellular matrix, nucleus and within intracellular compartments. Their secretion occurs via nonclassical pathways, often associated with oligomerization and membrane translocation. Functionally, galectins participate in cell–cell and cell–matrix interactions, regulation of cell growth and apoptosis, immune modulation, and pathogen recognition.^10,13–15^

Structurally, galectins share a conserved CRD of approximately 135 amino acids, arranged as a β-sandwich formed by two antiparallel β-sheets: one with six β-strands (S1–S6) and another with five (F1–F5).^16,17^ The highly conserved amino acids are located in β-strands S4–S6. The carbohydrate-binding site contains four principal subsites (A–D) and, in some cases, a fifth (E).^18^ Subsite C binds β-galactosides through highly conserved residues, while subsite D accommodates diverse saccharides, contributing to ligand specificity. The CRD contains highly conserved sequence motifs, including the heptapeptide HFNPRF, the tripeptide VCN, and the WGxExR motif, whose residues play central roles in galactose binding.^13,19^

Based on domain organization, galectins are classified into three structural types: prototype galectins, which contain a single CRD and form noncovalent dimers or tetramers; chimera-type galectins, which combine a C-terminal CRD with an N-terminal proline-rich domain that mediates oligomerization; and tandem-repeat galectins, which harbor two distinct CRDs connected by a linker peptide (Figure 1), enabling multivalent and heterobifunctional glycan recognition.^13,14^

**Figure 1:**
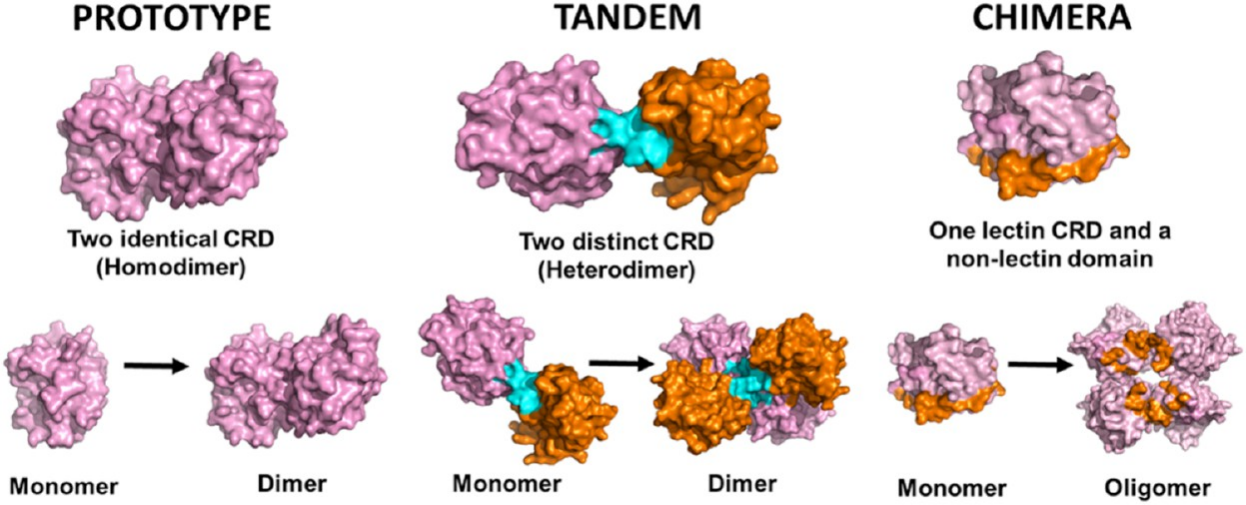
Structural classification of galectins. Prototype: Gal-1 used as model (1GZW). Tandem: Gal-8 used as model (4FQZ, dimer 4HAN). Chimera: Gal-3 used as model (1A3K, oligomer 6FOF).

## 3 Sponge Galectins

The interest in sponge lectins first emerged from the ability of their extracts to agglutinate erythrocytes, with first report of the presence of lectins in these organisms made in *Cliona celata* and *Axinella* sp. extracts.^20^

To date, more than 40 lectins have been isolated from sponges, most of them belonging to the class Demospongiae, which attracted greater attention because its environmental biomass is more compatible with lectin purification process. Moreover, this class comprises the largest number of sponge species and is predominantly found in shallow waters, factors that further facilitate their collection and experimental investigation. Only one lectin has been isolated from a Hexactinellida sponge^21^ and one from a Calcarea sponge.^22^

Structural information is available for a limited subset of sponge lectins, including galectins, C-type lectin, tachylectins, and unclassified (“orphan”) lectins from *Axinella polypoides* and *Haliclona caerulea*.^23–25^

Sponge galectins exhibit distinct structural and functional features compared with other animal galectins. They can form large, Ca^2+^-dependent molecular complexes, although calcium does not participate in carbohydrate binding, and display stronger affinity for GalNAc residues.^26,27^

Currently, galectins have been described in eight sponge species: three isolectins from *Geodia cydonium*,^28^ two from *Suberites domuncula*,^29,30^ one from *Halichondria okadai*,^31,32^ six isolectins from *Cinachyrella* sp.,^33^ one from *Chondrilla caribensis*,^34^ two isolectins from *Aplysina lactuca*,^35^ two isolectins from *Chondrilla australiensis*,^36^ and one from *Aiolochroia crassa*.^37^

In addition to experimentally characterized sponge galectins, several sequences annotated as galectins or galectin-like proteins are currently available in public databases such as UniProt and LectomeXplore. These entries are largely based on sequence similarity and domain prediction rather than direct biochemical or structural validation. Accordingly, this review focuses on sponge galectins with experimental biochemical and structural characterization, ensuring that the discussed properties are supported by direct evidence. In the following sections, each of these sponge galectins is described individually.

### 3.1 Structural Features of Sponge Galectins

This section summarizes the main structural and biochemical characteristics of galectins described so far from marine sponges. The sponge galectins characterized to date differ in molecular mass, oligomeric state, and carbohydrate specificity. In addiction, they share the conserved β-sandwich fold and CRD motifs typical of vertebrate galectins. Comparative analyses of their primary and predicted three-dimensional structures have revealed unique molecular adaptations, such as the extended loop forming a structured subsite E.

#### 3.1.1 *Geodia Cydonium* Galectin – First Sponge Galectin

In 1993, two lectins from the marine sponge *G. cydonium*, GcG-1 and GcG-2, were characterized at the molecular level using cDNA libraries. Two distinct genes, LECT-1 and LECT-2, were identified, encoding proteins of approximately 15 and 13–16 kDa, respectively. LECT-1 comprises 423 nucleotides encoding 140 amino acids, while LECT-2 contains 426 nucleotides encoding 141 amino acids, with a potential N-glycosylation site at Asn^126^.^28^

The two genes share 79% nucleotide and 65% amino acid identity, with 83% and 59% similarity in their 5′- and 3′-UTRs, respectively. Only LECT-1 encodes a cysteine residue. Both isolectins possess 37 conserved amino acids characteristic of the galectin CRD, including the canonical [LHFNPR­(G)­VNT­(G)­WT­(E)­EPF] motif. Their molecular weights, absence of proline/glycine-rich domains, and dimerization capacity support their classification as prototype galectins.^28^

No LECT-2 gene was detected in genomic DNA, confirming the absence of an alternative locus. Moreover, no phosphorylation or glycosylation was observed, contradicting earlier predictions.

The isoforms share high similarity with hamster galectins-1 and −3, though four of 15 conserved CRD residues (positions 22, 67, 71, 80) are substituted. Notably, Arg^71^→His^71^ directly affects carbohydrate recognition. The conserved motif DXAXHFNPR­(Y) was identified between residues 40–49, with two modified CRD regions: ^66^WGXEXR^71^→WQXEXH^66^ and ^75^FPFxxG^80^→ FPFxxN. Comparative alignments indicate that GcGs exhibit features intermediate between prototype and chimera-type galectins.^38^

#### 3.1.2 *Suberites domuncula* galectins

A galectin from the marine sponge *S. domuncula*, designated GALEC1_SBDO, was first characterized from a cDNA library. The protein comprises 193 amino acids, with a calculated molecular mass of 22.1 kDa, and contains a conserved galectin domain spanning residues Pro^5^-Gly^101^. The canonical HFNPR motif typical of galectins appears as ^24^HCNPR^28^ due to the substitution Phe^25^→Cys^25^.^30^

A second galectin, GALEC2_SBDO, was later identified using differential display and cDNA library approaches. It encodes 293 amino acids and contains two galactoside-binding domains: the first from Met^1^-Asp^117^ and the second from Glu^118^-Leu^293^. A hydrophobic segment occurs between Leu^275^–Leu^293^. In the second CRD, the conserved HFNPR sequence is replaced by ^183^HIGVR^187^. These two distinct CRDs, together with the C-terminal hydrophobic region, classify GALEC2_SBDO as a tandem-repeat galectin.^29^

#### 3.1.3 *Cinachyrella* Sp. Galectins

Two isolectins from the marine sponge *Cinachyrella* sp., CchG-1 and CchG-2, were characterized through N-terminal sequencing and Edman degradation of tryptic peptides. The resulting fragments were used to design primers, and the full-length cDNA sequences were obtained via RACE (Rapid Amplification of cDNA Ends). Multiple cDNA clones encoded a 147-amino-acid protein sharing 16% identity with eel congerin-I, 19% with human galectin-1, and 23% with the *G. cydonium* galectin.^33^

Five residues conserved in the CRD of the galectin family: Asn^60^, Trp^68^, Glu^71^, Arg^70^, and Arg^73^ were identified in CchG, supporting its classification within the prototype galectin subfamily.^33^

#### 3.1.4 Chondrilla caribensis

In 2021, the primary structure of the lectin from the marine sponge *C. caribensis* (CCL) was elucidated. CCL consists of 142 amino acids, with a calculated molecular mass of 15.4 kDa. No cysteine residues or glycosylation sites were identified, but microheterogeneity were observed, suggesting the presence of multiple isoforms.^34^

CCL exhibits a galectin-like domain spanning residues Tyr^2^-Ala^129^ and shares sequence similarity with an uncharacterized protein from *Amphimedon queenslandica*, a galectin from the nematode *Strongyloides ratti*, and a galectin-like protein from the amphibian*Rhinatrema bivittatum*. Conserved motifs typical of galectins were identified, including ^39^LHFNPRF^45^, ^62^VLN^64^, ^71^WG^72^, E^74^, R^76^, ^80^FPF^82^, and G^85^, with minor substitutions. Overall, the conservation of these residues and the similarity to other sponge and vertebrate galectins support the classification of CCL as a prototype galectin.^34^

#### 3.1.5 Aplysina lactuca

The galectin from the marine sponge *A. lactuca* (ALL) was characterized by,^35^ who determined the primary structures of two isoforms, ALL-a and ALL-b, each consisting of 144 amino acids.

Both isoforms contain three cysteine residues (Cys^12^, Cys^91^, Cys^136^) and a typical galectin domain architecture, extending from ^3^Ile-Val^138^ in ALL-a and ^3^Ile-Arg^133^ in ALL-b. The CRD is conserved, with minor sequence variation between the isoforms. Comparative analyses indicate that ALL shares strong similarity with CCL, supporting its classification as a prototype galectin.^35^

#### 3.1.6 Chondrilla australiensis

The primary structure of the lectin from the marine sponge *C. australiensis* (hRTL) was determined by Edman degradation and transcriptome analysis. RNA sequencing allowed identification of the transcript encoding the protein.^36^

The N-terminus of hRTL was unblocked, confirming that it corresponded to a mature form of the protein and enabling validation of transcriptomic data. RNA sequencing and tBLASTn searches identified six candidate sequences (hRTL-P1 to hRTL-P6), each beginning 23 residues upstream of the experimentally determined N-terminus, indicating the presence of a signal peptide cleaved post-translationally. The mature protein thus lacks N-terminal acetylation.^36^

All six sequences share high similarity among themselves and with the CCL. Isoforms hRTL-P1 and hRTL-P2 contain a 10-amino acid insertion identical to that of CCL. Both hRTL-P1 and hRTL-P2 retain the conserved residues required for β-galactoside binding, confirming that hRTL is a member of the prototype galectin family.^36^

#### 3.1.7 Aiolocroia crassa

The galectin from the marine sponge *A. crassa* (AcrL) was purified and characterized by Torres and colleagues.^34^ Its primary structure, comprising 146 amino acids. The sequence contains three cysteine residues; however, native ESI–MS revealed an apparent mass of ∼19.9 kDa, with no change after iodoacetamide alkylation. This result indicates the absence of reactive thiols and suggests the presence of post-translational modifications contributing to the observed mass increase.^37^

AcrL exhibits a canonical galectin domain extending from ^2^Leu-Val^140^, containing conserved CRD residues: His^40^, Asp^42^, Arg^44^, Asn^64^, Trp^73^, Glu^76^, and Arg^78^. Sequence alignment showed strong similarity to sponge galectins, including ALL-a and ALL-b and CCL. The conservation of CRD residues and overall sequence identity firmly place AcrL within the prototype galectin.^37^

#### 3.1.8 Halichondria okadai

The primary structure of the lectin from the marine sponge *H. okadai* (HOL-30) was determined through a combination of Edman degradation and transcriptome analysis. A tBLASTn search identified a single complete sequence showing high identity with peptides previously obtained by Edman degradation.^31,32^

HOL-30 lacks a signal peptide, and Edman degradation revealed a blocked N-terminus. The protein comprises 281 amino acids with a calculated molecular mass of 30.8 kDa. Structurally, HOL-30 is a tandem-repeat galectin, containing two CRDs that form a noncovalent dimeric organization.^31^

Sequence analysis showed moderate identity with *S. domuncula* galectin-2. In both proteins, the linker peptide between the two CRDs is extremely short, nearly absent. The key residues involved in carbohydrate recognition are conserved in both domains. Unlike *S. domuncula* galectin-2, HOL-30 lacks the C-terminal hydrophobic tail and contains no cysteine residues.^31^

### 3.2 Structural Diversity and Functional Implications of Sponge Galectins

Galectins typically lack native disulfide bonds, but cysteine residues, when present, can participate in redox-sensitive conformational changes.^13,29,39^ Considering sponges, CCL, HOL-30 and hRTL-P1 lack cysteines, while others (e.g., *Cinachyrella* sp.) display cysteine-dependent structural modulation.^31,34,36,40^ In ALL, redox conditions regulate oligomerization: tetrameric under reducing, multimeric under oxidizing conditions.^35^

A blocked N-terminus, often acetylated, is common in animal galectins but absent in CCL, ALL and hRTL-P1.^34–36^ Interestingly, *G. cydonium* and *C. australiensis* galectins contain a signal peptide, an uncommon feature in this family that is generally secreted via nonclassical pathways.^36,41^

Sponge galectins exhibit the canonical domain seen in other metazoans. Most belong to the prototype class (single CRD), including CchGs, CCL, ALLs, hRTL, and AcrL.^33,34,36,37,42^ In contrast, HoL-30 is tandem-type, while CGC combines prototype structure with chimera-like binding properties.^38^ The galectin Sd 1 from *S. domuncula* can be classified into a prototype and the Sd 2 galectin into a tandem type.^29^

The main characteristics observed in these galectins are summarized in Table 1.

**Table 1:** Characteristics of Primary Structures from Sponge Galectins^a^

sponge	galectin	number of amino acids	number of cysteines	type	accession number
*A. crassa*	AcrL	146	3	proto	N/d
*A. lactuca*	ALL-a	144	3	proto	C0HM19.1
*A. lactuca*	ALL-b	144	3	proto	C0HM20.1
*Cinachyrella sp.*	CchG1	146	2	proto	4AGV_D
*C. caribensis*	CCL	142	-	proto	C0HLX7.1
*S. domuncula*	GALEC1_SBDO	193	6	proto	CAD37940.1
*S. domuncula*	GALEC2_SBDO	293	6	tandem	CAJ43112.1
*G. cydonium*	GCG_13 kDa	141	1	proto/chimera	CAA50198.1
*G. cydonium*	GCG_15 kDa	140	1	proto/chimera	N/d
*G. cydonium*	GCG_16 kDa	140	1	proto/chimera	N/d
*H. okadai*	HOL-30	281	-	tandem	XHV10860.1
*C. australiensis*	hRTL-P1	141	-	proto	N/d
*C. australiensis*	hRTL-P2	141	1	proto	N/d

a N/d - sequences not deposited in public databases, available in references 31,37,38

The conserved sequence galectin motifs “LHFNPRF,” “VCN,” “WGXEXR,” and “FPF” are all present in sponge galectins but display consistent residue substitutions. For instance, in the heptapeptide “LHFNPRF,” only Arg remains conserved across all sponge sequences. The tripeptide “VCN” typically mutates to “ILN” or “VLN,” and in “WGxExR,” Trp and Glu remain largely preserved (Figure 2).

**Figure 2:**
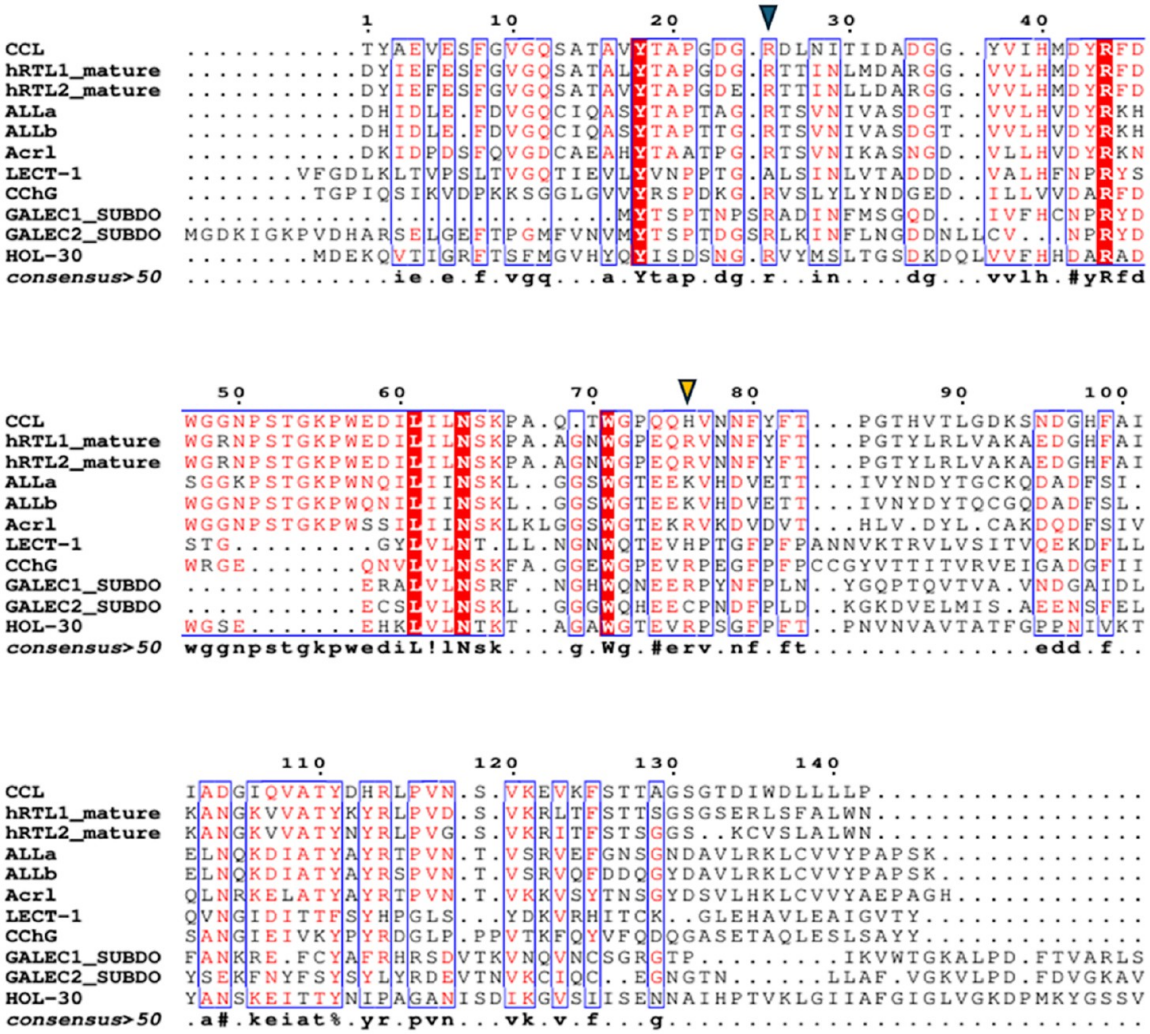
Alignment of sponge galectins. CCL from *C. caribensis* (C0HLX7.1); hRTL-1 and hRTL-2 from *C. australiensis*;^31^ ALL from *A. lactuca* (C0HM19.1; C0HM20.1). Acrl from *A. crassa*.^34^ LECT-1 from *G. cydonium* (CAA63818.1). CchG from *Cinachyrella* sp. (4AGR). HOL-30 from *H. okadai* (XHV10860.1). GALEC1_SUBDO and GALEC2_SUBDO from *S. domuncula* (CAD37940.1; CAJ43112.1). Amino acid numbering is based on the CCL sequence. The C-terminal regions of *S. domuncula* galectins were excluded for improved visualization and interpretation. Dots above the alignment indicate intervals of ten residues, and dots within sequences represent alignment gaps. The blue inverted triangle indicates the beginning of the CRD, considering all sponge galectins with predicted analyses based on molecular docking. The orange inverted triangle indicates the end of the CRD. Red residues indicate conserved or highly similar amino acids, whereas nonhighlighted residues correspond to variable positions. Blue boxes indicate structurally conserved regions.

Sponges exhibit unique conservation patterns, including Arg of the heptapeptide (LHFNPRF), Asn of the tripeptide “VCN”, and Trp of the domain “WGxExR”. Asp/Asn preceding the heptapeptide is another sponge-conserved feature. The residues Pro^21^ and Tyr^18^, using the CCL sequence as a reference, can also be considered conserved in sponge galectins, as they are present in all sequences with the exception of tyrosine in HOL-30 and proline in HOL-30 and AcrL.

A conserved sequence segment, NPSTGKPWEDI, was identified exclusively in galectins from *C. caribensis*, *C. australiensis*, *A. lactuca* and *A. crassa*. This motif is absent from other sponge galectins and appears in a region adjacent to the canonical CRD. The conservation of this sequence among phylogenetically related demosponges may reflect a lineage-specific structural adaptation, potentially associated with glycan-recognition fine-tuning or protein–protein interactions that stabilize the CRD conformation. Its restriction to these species suggests a divergent evolutionary path within the Demospongiae galectin repertoire.

Sponge galectins retain several residues conserved in vertebrates (e.g., L/I^61^, using the CCL sequence as a reference and K flanking “VCN”) but absent in other invertebrate phyla. Comparative analyses^34^ reveal that sponge galectins share more conserved features with vertebrate than with invertebrate galectins such as those from nematodes, mollusks, or echinoderms, suggesting an early evolutionary stabilization of core structural elements (Figure 3).

**Figure 3:**
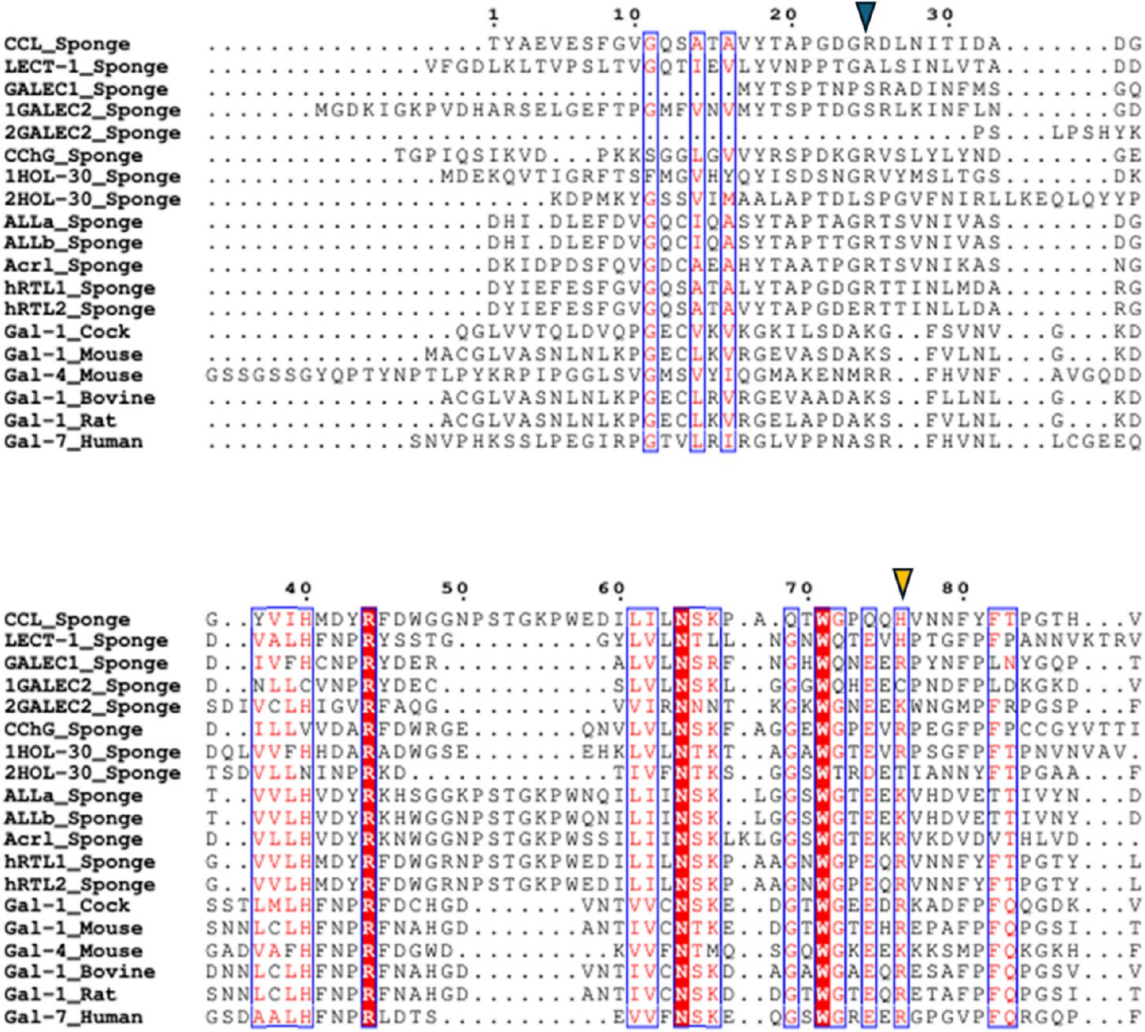
Alignment of sponge and vertebrate galectin: conserved sequence motifs. CCL (C0HLX7.1); hRTL-1 and hRTL-2;^31^ ALLs (C0HM19.1; C0HM20.1); Acrl;^34^ LECT-1 (CAA63818.1); CchG (4AGR); 1HOL-30 and 2HOL-30 correspond to the two domains of the HOL-30 galectin (XHV10860.1); GALEC1 (CAD37940.1), 1GALEC2 and 2GALEC2, correspond to the two domains of the GALEC2 galectin (CAJ43112.1); Gal-1_Cock from *Gallus gallus* (1QMJ_1); Gal-1_Mouse and Gal-4_Mouse from *Mus musculus* (4LBQ_A; 2DYC_1); Gal-1_Bovine from *Bos taurus* (1SLA_1); Gal-1_Rat from *Rattus norvegicus* (4GA9_1); Gal-7_Human from *Homo sapiens* (4GAL_1). Amino acid numbering is based on the CCL sequence. Dots above the alignment indicate intervals of ten residues, and dots within sequences represent alignment gaps. The C-terminal regions of galectins were excluded for improved visualization and interpretation. The blue inverted triangle indicates the beginning of the CRD, considering all sponge galectins with predicted analyses based on molecular docking. The orange inverted triangle indicates the end of the CRD. Red residues indicate conserved or highly similar amino acids, whereas nonhighlighted residues correspond to variable positions. Blue boxes indicate structurally conserved regions. The selection of vertebrate galectins included in the alignment was based on the availability of three-dimensional structures determined by X-ray crystallography. The domains of tandem-type galectins were separated to improve visualization of the conserved sequence. The animal group to which each galectin belongs is indicated after the underscore (_).

According to^34^ there are many changes in the conserved residues of the conserved domains of sponge galectins with galectins from other invertebrates, mainly in the main heptapeptide (Figure 4). In fact, only the galectins from the snail *Biomphalaria glabrata* (BgGal), the nematode *Toxascaris leonina* and the echinoderm *Apostichopus japonicus* possess the conserved vertebrate heptapeptide: LHFNPRF. The tripeptides VCN and FPF suffered alterations in most sequences, where the first Phe is altered only in the nematode *T. leonina* and in the sponge galectins ALLs and AcrL.

**Figure 4:**
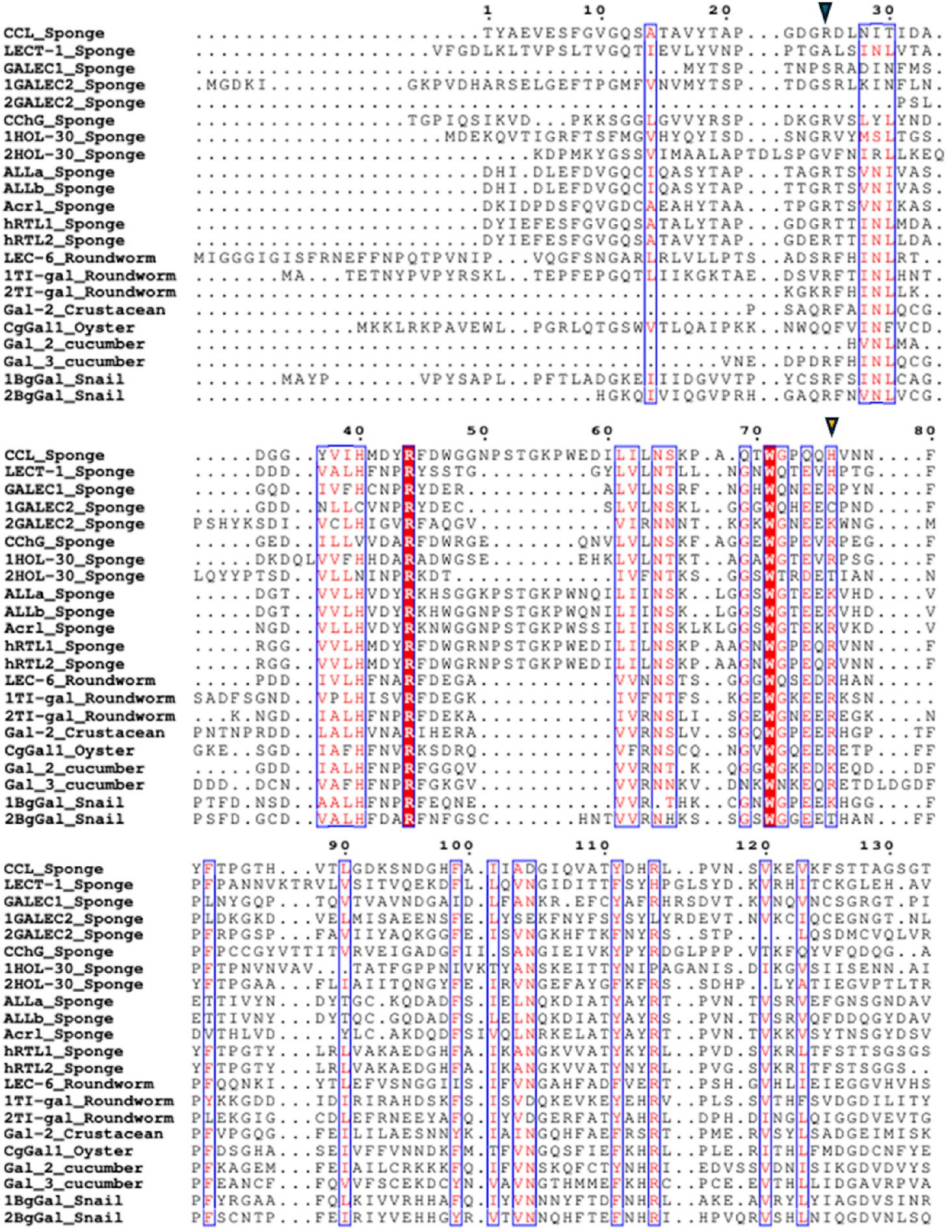
Alignment of sponge and invertebrate galectins: conserved sequence motifs. CCL (C0HLX7.1); hRTL-1 and hRTL-2;^31^ ALLs (C0HM19.1; C0HM20.1); Acrl;^34^ LECT-1 (CAA63818.1); CchG (4AGR); 1HOL-30 and 2HOL-30 correspond to the two domains of the HOL-30 galectin (XHV10860.1); GALEC1 (CAD37940.1), 1GALEC2 and 2GALEC2, correspond to the two domains of the GALEC2 galectin (CAJ43112.1); LEC-6_Roundworm from *Caenorhabditis elegans* (3VV1_1); 1TI-gal and 2TI-gal_Roundworm correspond to the two domains of the TI-gal from *T. leonina* (4HL0_1); Gal-2_crustacean from *Daphnia pulex* (AFI40275.1); CgGal1_Oyster from *Crassostrea gigas* (BAF75419.1); Gal_2 and Gal_3_cumcumber correspond to the two domains of the galectin from sea cumcumber *A. japonicus* (PIK56913.1); 1BgGal and 2BgGal_Snail correspond to the two domains of the BgGal from from *B. glabrata* (ABQ09359.1). Amino acid numbering is based on the CCL sequence. Dots above the alignment indicate intervals of ten residues, and dots within sequences represent alignment gaps. The C-terminal regions of galectins were excluded for improved visualization and interpretation. The blue inverted triangle indicates the beginning of the CRD, considering all sponge galectins with predicted analyses based on molecular docking. The orange inverted triangle indicates the end of the CRD. Red residues indicate conserved or highly similar amino acids, whereas nonhighlighted residues correspond to variable positions. Blue boxes indicate structurally conserved regions. The selection of invertebrate galectins included in the alignment was based on the availability of three-dimensional structures determined by X-ray crystallography, in the absence of experimentally determined three-dimensional structures, primary sequences were used. The domains of tandem-type galectins were separated to improve visualization of the conserved sequence. The animal group to which each galectin belongs is indicated after the underscore (_).

Threrefore, independent of phylum the only residues that are fully preserved among the vast majority galectins, are Arg (LHFNPRF), Trp and Glu (WGxExR) (Figure 5). Even though Glu only changes in CCL, this residue is present in all sequences. The Arg residue (WGxExR) was shown to be highly preserved only in vertebrate galectins, while among invertebrates it is not a perfectly conserved amino acid.

**Figure 5:**
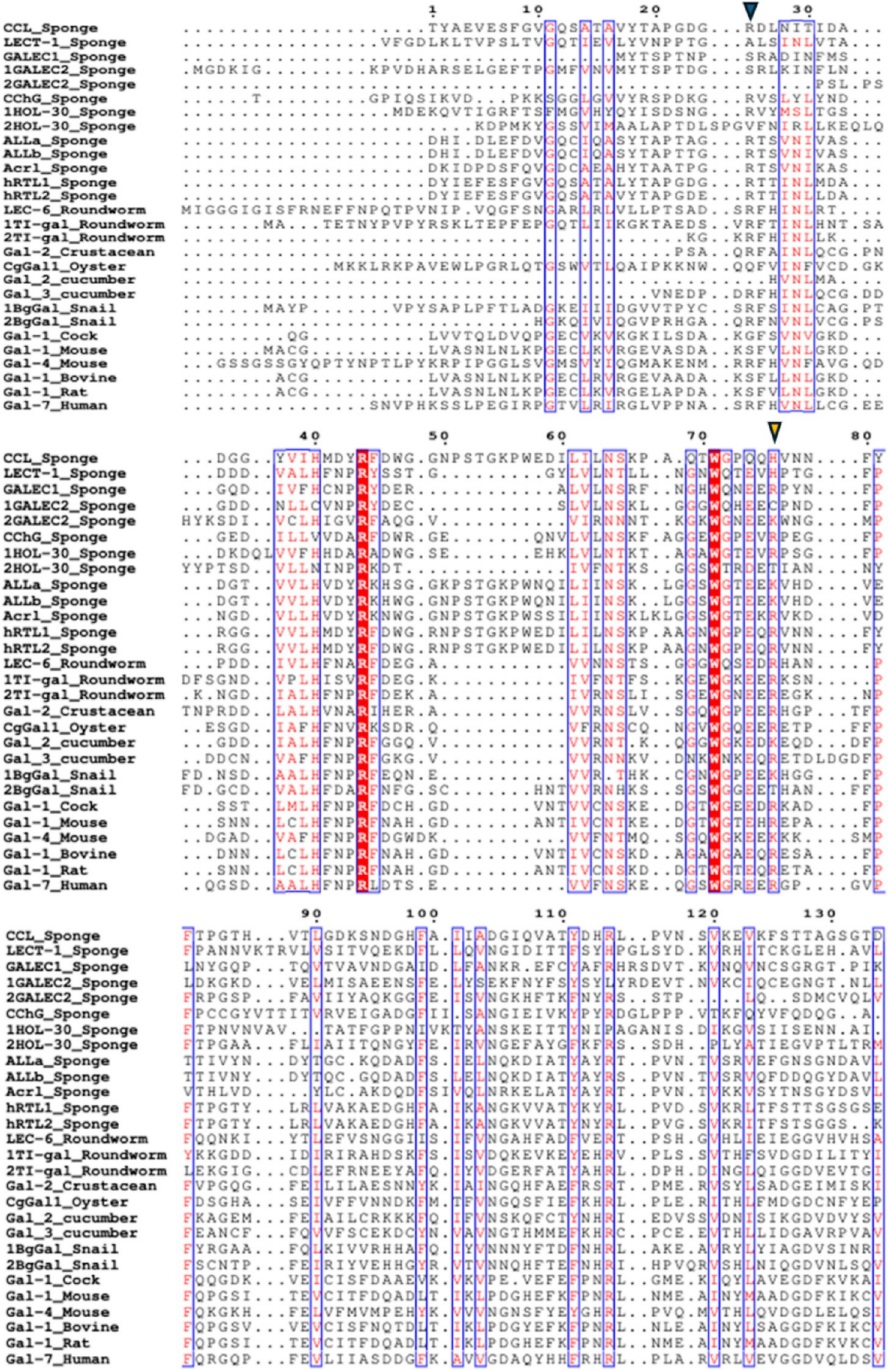
Alignment of invertebrate and vertebrate galectins: conserved sequence motifs. References indicated in Figures 3 and 4. Amino acid numbering is based on the CCL sequence. Dots above the alignment indicate intervals of ten residues, and dots within sequences represent alignment gaps. The C-terminal regions of galectins were excluded for improved visualization and interpretation. The blue inverted triangle indicates the beginning of the CRD, considering all sponge galectins with predicted analyses based on molecular docking. The orange inverted triangle indicates the end of the CRD. Red residues indicate conserved or highly similar amino acids, whereas nonhighlighted residues correspond to variable positions. Blue boxes indicate structurally conserved regions. The selection of galectins included in the alignment was based on the availability of three-dimensional structures determined by X-ray crystallography, in the absence of experimentally determined three-dimensional structures, primary sequences were used. The domains of tandem-type galectins were separated to improve visualization of the conserved sequence. The animal group to which each galectin belongs is indicated after the underscore (_).

Nevertheless, NPSTGKPWEDI sequence appears to be exclusive to sponge galectins, as it is not observed in vertebrate or invertebrate galectins from other phyla.

### 3.3 Three-Dimensional Structure and Structural Models of Sponge Lectins

While numerous three-dimensional structures of animal galectins have been experimentally determined, sponge galectins remain a notable exception. To date, only the galectin CchG-1 from *Cinachyrella* sp. has had its three-dimensional structure solved by X-ray crystallography. In contrast, the structural information available for CCL, ALL-a, hRTL, AcrL and HOL-30, is based on computational approaches, including homology modeling and molecular docking, derived from experimentally solved galectin templates.^34–37,40^

All sponge galectins characterized so far either experimentally or through validated homology models appear to share the canonical β-sandwich architecture, composed of two antiparallel β-sheets surrounding a hydrophobic core, which represents a defining structural hallmark of the galectin family. CchG-1, the only crystallographically resolved sponge galectin, is a canonical dimer whose subunits align antiparallelly, stabilized by an unusual intersubunit disulfide bridge, an exclusive feature among known galectins (Figure 6). Its was crystallized in the absence of carbohydrate ligands. CRD was identified based on the conserved galectin β-sandwich fold and structural superposition with ligand-bound galectins, allowing the binding site to be inferred from conserved residues. The CRD comprises the conserved β5-strand motif ^53^EQNVLVLNS^61^, central to carbohydrate binding, with key residues Arg^28^, Arg^47^, Asn^60^, Trp^68^, and Glu^71^ mediating interactions analogous to those seen in vertebrate galectins^40^ (Figure 7B).

**Figure 6:**
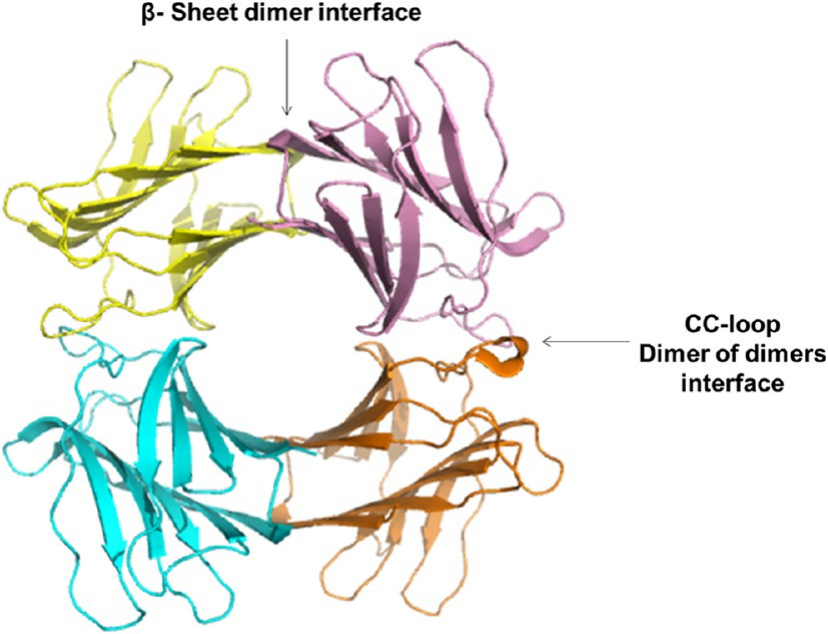
CchG crystal structure. Adapted from.^42^ Tape representation of the CchG tetramer, where two interfaces mediate the dimer of dimers. The location of the two interfaces is marked. Protein Data Bank (PDB) access code 4AGV.

**Figure 7:**
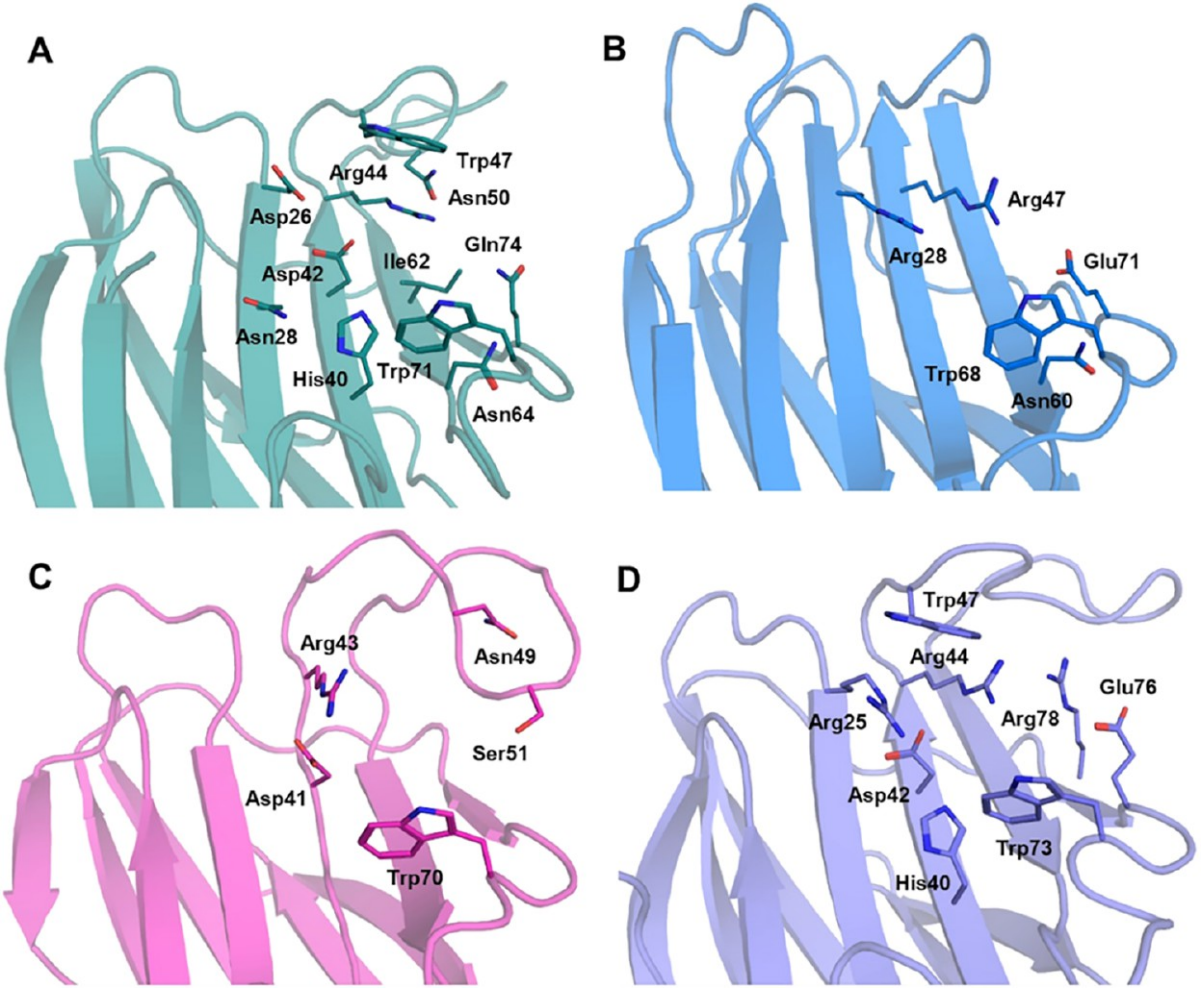
Comparative analysis of marine galectins carbohydrate recognition domains (CRD). (A): CCL; (B): CchG; (C): ALL-b; (D): AcrL. The protein structures are represented as colored cartoons and the interacting residues represented as colored lines. The amino acids involved in the carbohydrate-binding site are consistent with those predicted by molecular analyses.^42,31,32,34^

Homology-based structural models for other sponge galectins suggest a remarkable conservation of the β-sandwich topology but reveal subtle lineage-specific features (Figura [Fig fig8]). CCL structural model lacks cysteines and disulfide bridges and displays a broad, shallow binding cavity capable of accommodating elongated glycans. The carbohydrate-binding site, predicted by molecular docking, includes residues D^26^, N^28^, H^40^, D^42^, R^44^, W^47^, N^50^, I^62^, N^64^, W^71^, and Q^74^ arranged over the curved β-sheet typical of prototype galectins (Figure 7A).^34^ Its dimeric organization predicted by molecular analyses is conserved among sponges, whereas the tetrameric interface predicted for CCL resembles that found in *Salmo salar* galectin, suggesting ancient conservation of quaternary packing.

**Figure 8:**
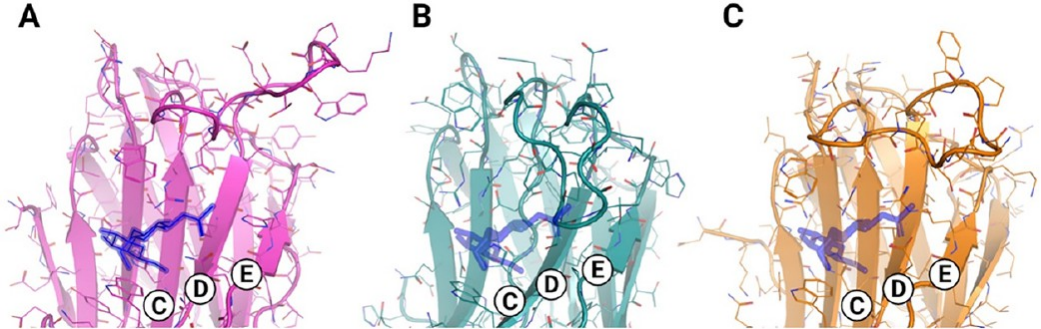
Homology-based three-dimensional models of the carbohydrate-binding region in sponge galectins. Comparison of the canonical β-sandwich fold highlighting subsites C, D, and E in three representative sponge galectins: (A) *C. caribensis* (CCL), (B) *A. lactuca* (ALL), and (C) *A. crassa* (AcrL). All structures display the conserved β-strands forming subsites C and D, while a distinctive and well-defined loop gives rise to an extended and structured subsite E. This subsite, which is poorly defined in most vertebrate galectins, is particularly prominent in sponge galectins and likely contributes to their broader glycan-binding spectrum and higher affinity for large and sialylated carbohydrates. The region highlighted with a thicker line corresponds to the loop motif WGRNPSTGKPW.

Structural models of the isolectins ALL-a and ALL-b maintain the same β-sandwich framework, with minor variations in strand and loop numbers (Figures 7C and 8B). Both feature an extended β4−β5 region that defines the CRD, engaging lactose via hydrogen bonds and van der Waals contacts with residues D^41^, R^43^, T^52^, and W^70^ in ALL-a, and D^41^, R^43^, N^49^, S^51^, and W^70^ in ALL-b.^35^ Similarly, homology-based structural models of AcrL indicate a compact dimeric arrangement in which the ^49^NPSTGKPW^57^ loop extends the CRD and enlarges the glycan-binding pocket. Molecular docking suggests that this loop may facilitate the accommodation of bulky and sialylated oligosaccharides, such as fetuin (Figure 8C). These interactions, involving R^25^, H^40^, D^42^, R^44^, W^47^, W^73^, E^76^, and R^78^, highlight an expanded structural versatility among demosponges.^37^

A comparable loop motif, WGRNPSTGKPW, also characterizes the structural model of the hRTL lectins further suggesting a lineage-specific structural signature among Chondrillidae and Verongimorpha species.^36^ In these models, the elongated loop appears to project outward from the β-sandwich, reshaping the CRD and forming additional hydrogen bonds with the TF antigen, thereby fine-tuning carbohydrate affinity. The recurrence of this loop across phylogenetically related sponges supports the hypothesis that it emerged as a functional adaptation for binding more complex marine glycans.

The tandem-type HOL-30 deviates from the prototype architecture by containing two CRDs arranged in series. As established by structural models, each domain appears to retain the galectin β-sandwich topology, with moderately conserved glycan-binding residues, but the linker connecting them is extremely short, suggesting restricted interdomain flexibility. Structural superposition with human galectin-1 and hRTL indicates overall conservation of the β-core but absence of the accessory loop characteristic of the Chondrilla-type galectins.^31^

Altogether, structural analyses of sponge galectins suggest a consistent β-sandwich fold across all species, accompanied by distinct innovations in loop architecture and quaternary assembly. The CRD is the functional core of galectins: saccharide-binding specificity, affinity, and conformational flexibility arise from subtle variations in the β-sandwich topology and the length and dynamics of connecting loops that shape the binding pocket. Despite this overall conservation, sponge galectins display unique loop extensions that remodel the canonical CRD and expand its recognition surface toward larger and more complex glycans.

A particularly striking feature is the NPSTGKPWEDI motif, recurrent in *Chondrilla*, *Aplysina*, and *Aiolochroia* galectins, which likely represents an evolutionary expansion of the CRD. In canonical galectins, the so-called subsite E is usually weakly structured and provides limited interaction with moieties extending from the reducing end of saccharides bound at subsites C and D.^43^ However, in these sponge galectins, the elongated loop containing NPSTGKPWEDI appears to form a well-structured and stable subsite E, capable of establishing additional hydrogen bonds and hydrophobic contacts that broaden the CRD and accommodate bulkier or branched glycans, including sialylated termini. This structural reinforcement likely underlies the enhanced affinity and broader ligand repertoire of marine galectins, suggesting that the loop extension represents an early evolutionary innovation that refined the carbohydrate-binding topology and expanded glycan-recognition capacity within the galectin family.

### 3.4 Structural Evolution of Sponge Galectins

Phylogenetic analyses position sponge galectins at the basal region of the galectin family tree (Figure 9), indicating that Porifera harbor the most ancestral representatives of this protein family. In the tree, sponge prototype galectins form the earliest-diverging cluster, whereas tandem-repeat galectins occupy more derived positions, consistent with the view that the duplication giving rise to bi-CRD galectins occurred after the emergence of the proto-type forms. Within Porifera, the tandem galectin HOL-30 exemplifies this evolutionary transition: its two CRDs share low sequence identity, reflecting divergent rather than simple duplicative evolution.^31^ These patterns support the notion that sponge galectins retain primitive structural and genetic features from which later invertebrate and vertebrate galectins emerged.

**Figure 9:**
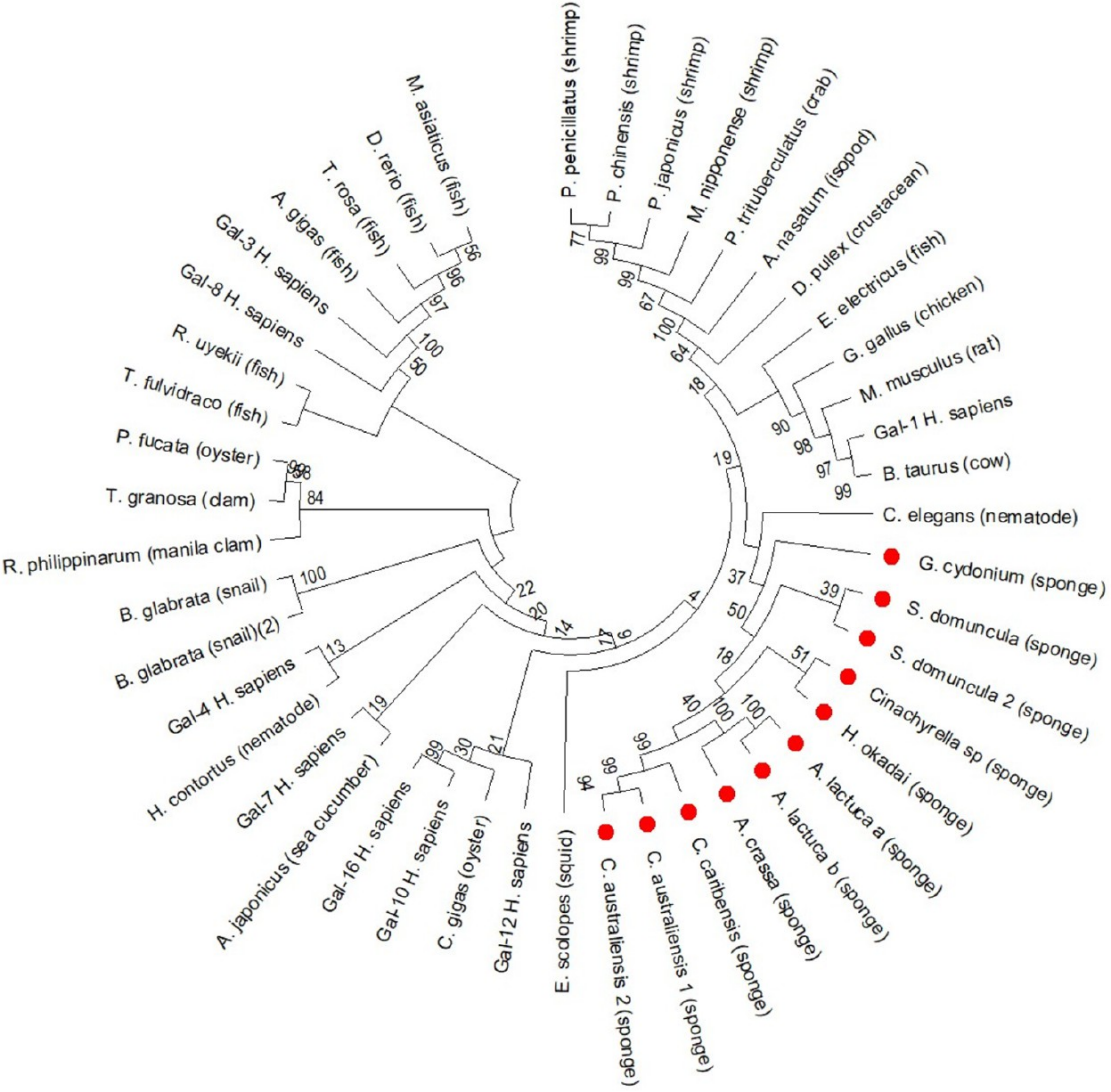
Phylogenetic tree of galectins from Porifera and representative invertebrate and vertebrate taxa. The tree was constructed using the Neighbor-Joining (NJ) method with 1000 bootstrap replicates; bootstrap support values (%) are indicated at the nodes. Sponge galectins are highlighted with red circles, allowing visualization of their basal and distinct clustering relative to other metazoan galectins. The topology supports the early divergence of Porifera galectins within the galectin family and the subsequent separation of invertebrate and vertebrate lineages.

Functional data corroborate this evolutionary interpretation. Several sponge galectins, including CCL, AcrL, and hRTL, exhibit the ability to bind complex glycans such as blood-group oligosaccharides and sialylated structures, activities typically associated with vertebrate tandem galectins, suggesting that broad glycan recognition and high-affinity binding likely originated early during metazoan evolution, preceding the diversification of bilaterian immune systems. The subsequent expansion of the galectin family appears to have paralleled the transition from simple innate immune responses to the more specialized and compartmentalized immune functions observed in vertebrates.^44^

Although sponge galectins cluster phylogenetically closer to other invertebrate homologues, several molecular characteristics reveal unexpected affinities with vertebrate galectins. For example, CCL appears to exhibit a rare tetrameric interface previously reported only for *Salmo salar* galectin-1,^34^ suggesting that similar quaternary arrangements may have evolved independently in distantly related lineages. Such convergence is well documented in other lectin families, including C-type and legume lectins, where unrelated evolutionary trajectories have produced comparable carbohydrate-binding architectures.^16,45,46^

From an evolutionary standpoint, sponges hold a pivotal position among metazoans: they are the earliest extant lineage possessing essential extracellular matrix components (e.g., collagen) and key signal-transduction systems required for multicellularity.^1^ The extracellular galectin from *G. cydonium*, which mediates cell adhesion, may therefore represent an ancestral prototype of the more functionally specialized galectins observed in complex animals.^38^ Given their basal phylogenetic position, structural simplicity, and functional versatility, sponge galectins are strong candidates for the ancestral form from which the entire galectin family evolved.

Thus, the molecular and structural diversity of sponge galectins illuminates the early evolutionary history of metazoan lectins and highlights the adaptive pathways that shaped carbohydrate recognition mechanisms across animal evolution.

### 3.5 Main Physiological Roles of Sponge Galectins

Sponge galectins play diverse physiological roles, primarily associated with cell–cell recognition, immune defense, and morphogenesis. In the absence of an adaptive immune system, these lectins participate in innate immune responses, mediating pathogen recognition through specific interactions with surface glycoconjugates.^47,48^

CCL, AcrL, ALLs, and HOL-30 exhibit antibacterial and antibiofilm properties.^34,35,37,49^ These findings suggest that sponge galectins contribute to the innate immune system by recognizing PAMPs and inhibiting microbial colonization.

Beyond immunity, galectins also appear to play roles in morphogenesis and skeletal formation. The *S. domuncula* galectin has been detected in the axial canal and on the surface of spicules, where it interacts with silicatein, a silica–polymerizing enzyme, enhancing its activity and contributing to spiculogenesis.^29^ Likewise, the HOL-30 localizes around spicule-forming tissues, reinforcing its involvement in biomineralization.^31^

GCG mediate cell adhesion through interactions between carbohydrate residues on the cell surface and those within the extracellular matrix, functioning as aggregation factors during tissue organization.^21,27^ Collectively, these observations highlight that sponge galectins, though structurally related to vertebrate counterparts, have evolved specialized physiological roles in immune recognition, morphogenesis, and skeletal development within the Porifera lineage.

### 3.6 Biological Activities of Sponge Galectins

Sponge galectins have gained attention as promising molecules for biomedical and biotechnological applications due to their low immunogenicity, small molecular size, and diverse biological activities.^48,50^ These proteins exhibit antibacterial, antibiofilm, cytotoxic, and neuromodulatory properties, many of which parallel or even extend the functional versatility of vertebrate galectins.

CCL, AcrL, and ALL show potent antibacterial and antibiofilm effects against *Staphylococcus aureus*, *S. epidermidis*, and *Escherichia coli*, suggesting potential applications in the development of antimicrobial agents.^37,51,52^ Importantly, some of these galectins exhibit synergistic effects with conventional antibiotics: ALL enhances the efficacy of ampicillin and oxacillin and AcrL acts synergistically with oxacillin.^35,37^ Such interactions highlight their potential use as adjuvants in therapies targeting multidrug-resistant bacteria.

CCL exerts pronounced anti-Leishmania infantum activity: it binds to parasite surface glycans via its CRD, disrupts membrane integrity, induces reactive oxygen species (ROS) generation, and triggers apoptotic death of promastigotes. This multistep mechanism underscores its biotechnological potential as an antiprotozoal agent.^34^

The galectin hRTL exhibits anticancer potential. This tetrameric galectin, which appears to retain the canonical β-sandwich fold and a distinctive elongated loop near its CRD, binds with high affinity to the Thomsen-Friedenreich (TF) antigen, sialyl-TF, and type-1 N-acetyl-lactosamine (Galβ1–3GlcNAc) glycans, glycan motifs commonly overexpressed in tumor cells. When tested against DLD-1 colorectal carcinoma cells, hRTL reduced cell growth at low concentrations.^36^

The galectins CchG-1 and CchG-2 were discovered by screening for modulation of mammalian ionotropic glutamate receptors: in electrophysiological assays, application of CchGs significantly slowed the decay phase of GluA4- and GluK2a-mediated currents in HEK293 cells, by a factor of 4–8×, indicative of a positive allosteric effect on AMPA and kainate receptor subtypes. In vivo, crude extracts containing CchGs induced convulsions in rats, implicating their interaction with central nervous system (CNS) glutamate signaling.^33^

Sponge galectins combine ancient immune functions with diverse biological properties that can be leveraged in modern biotechnology. Their selective carbohydrate recognition and ability to synergize with existing drugs position them as promising candidates for the development of novel antimicrobial, antiparasitic, and anticancer therapies, as well as molecular tools for studying cell communication and neural modulation.

## 4 Conclusions

Although vertebrate galectins, particularly those from mammals, have been extensively investigated, encompassing detailed knowledge of their structures, subcellular localization, and roles in signaling and immune modulation, the understanding of invertebrate galectins remains incipient. Among them, sponge galectins occupy a key phylogenetic position that bridges unicellular ancestors and multicellular metazoans. These primitive lectins retain the canonical β-sandwich fold and CRD motifs typical of vertebrate galectins yet display unique molecular innovations that expand our view of the family’s functional evolution.

Recent structural analyses revealed the presence of an extended loop shaping a well-defined subsite E in several sponge galectins, a region that is generally unstructured in vertebrate homologues. This additional pocket contributes to the binding of bulky and sialylated glycans and may explain the higher affinity and broader specificity observed in these proteins. Such an architectural innovation suggests that poriferan galectins have independently evolved mechanisms to interact with complex carbohydrates, possibly anticipating molecular solutions later refined in vertebrates.

Functionally, sponge galectins participate in innate defense, morphogenesis, and, remarkably, in interactions with mammalian targets such as neural glutamate receptors and tumor-associated glycans, revealing their potential as molecular models and biotechnological tools. Phylogenetic and structural data consistently indicate that the prototype galectin fold emerged early in Metazoan evolution, and that subsequent domain duplication and divergence led to the diversity observed in higher animals.

Therefore, the study of sponge galectins provides a unique window into the early molecular evolution of glycan-binding proteins. By integrating structural, functional, and evolutionary perspectives, these investigations help clarify the origins of animal galectins but also unveil new structural determinants that may be harnessed for biomedical and biotechnological applications.
